# Dapagliflozin improves cardiac function and reduces adverse events in myocardial infarction: a meta-analysis in diabetic and non-diabetic populations

**DOI:** 10.3389/fendo.2025.1594861

**Published:** 2025-06-04

**Authors:** Shuang Li, Longlong Wang, Peng Wang, Xiaohui Xu, Yanhua Guo

**Affiliations:** ^1^ Department of Cardiology, Qilu Hospital of Shandong University Dezhou Hospital, Dezhou, Shandong, China; ^2^ Department of Genetics and Cell Biology, School of Basic Medicine, Qingdao University, Qingdao, Shandong, China

**Keywords:** dapagliflozin, myocardial infarction, heart failure, meta-analysis, type 2 diabetes

## Abstract

**Background:**

Myocardial infarction (MI) remains a leading cause of morbidity and mortality worldwide, frequently driven by acute coronary occlusion resulting from atherosclerosis and arrhythmias. Type 2 diabetes mellitus (T2DM) is a major risk factor for atherosclerotic progression and is associated with worsened cardiovascular outcomes in post-MI patients. Dapagliflozin, a sodium-glucose co-transporter 2 (SGLT2) inhibitor, has emerged as a novel antidiabetic agent with additional cardiovascular benefits. Increasing evidence suggests its potential utility in post-MI care, particularly in patients with coexisting T2DM.

**Objective:**

This study aims to systematically evaluate the clinical efficacy of dapagliflozin in improving cardiac function and reducing adverse cardiovascular events in post- MI patients with and without diabetes.

**Methods:**

A systematic search of PubMed, Embase, Web of Science, Cochrane Library, CNKI, and WanFang databases identified relevant clinical studies up to May 22, 2024. Eligible randomized controlled trials (RCTs) and retrospective cohort studies were analyzed using Review Manager 5.3.

**Results:**

19 studies (12 RCTs and 7 cohort studies) with 7,128 patients were included. Meta-analysis showed dapagliflozin significantly reduced key cardiac biomarkers and structural parameters, including NT-proBNP (MD = -62.06, 95% CI [-94.59, -29.53], P = 0.0002), LVEDD (MD = -2.58, 95% CI [-3.64, -1.52], P < 0.00001), and LVESD (MD = -2.32, 95% CI [-2.99, -1.66], P < 0.00001), while enhancing LVEF (MD = 3.88, 95% CI [2.24, 5.52], P < 0.00001). It also reduced major adverse cardiovascular events (RR = 0.33, 95% CI [0.18, 0.60], P < 0.05), and heart failure-related rehospitalization (RR = 0.53, 95% CI [0.30, 0.91], P < 0.05). Subgroup analysis revealed consistent cardioprotective benefits in both diabetic and non-diabetic populations.

**Conclusion:**

Dapagliflozin significantly enhances cardiac function and reduces adverse cardiovascular events in post-MI patients, independent of diabetes status. These findings support the integration of dapagliflozin into post-MI management strategies. Further large-scale, long-term clinical trials are needed to assess its impact on recurrent MI and long-term survival outcomes.

## 1 Introduction

Myocardial infarction (MI), commonly referred to as a heart attack, results from the acute obstruction of coronary arteries, primarily due to atherosclerotic plaque rupture and subsequent thrombosis. This ischemic event leads to cardiomyocyte necrosis and apoptosis, initiating a cascade of inflammatory and fibrotic responses that promote ventricular remodeling and progressive cardiac dysfunction (1). Clinically, MI manifests as severe, prolonged chest pain, often accompanied by autonomic symptoms such as nausea, dizziness, and diaphoresis (2). Despite substantial advancements in reperfusion strategies, including thrombolysis, percutaneous coronary intervention (PCI), and coronary artery bypass grafting (CABG), MI remains a leading cause of global morbidity and mortality, as post-MI patients continue to face a high risk of recurrent cardiovascular events and heart failure progression (3–5).

Of particular note, among MI patients, individuals with type 2 diabetes mellitus (T2DM) exhibit disproportionately higher rates of adverse cardiovascular outcomes (6, 7). Mechanistically, hyperglycemia and insulin resistance drive endothelial dysfunction, oxidative stress, and chronic low-grade inflammation, exacerbating atherosclerosis and increasing plaque vulnerability (8–10). Consequently, diabetic patients not only experience a heightened risk of MI but also demonstrate impaired myocardial healing, adverse ventricular remodeling, and increased long-term mortality compared to non-diabetic individuals (11, 12). Given the pathophysiological interplay between glucose dysregulation and cardiovascular disease, targeting metabolic pathways has emerged as a promising strategy for improving post-MI outcomes.

Sodium-glucose cotransporter 2 inhibitors (SGLT2i) are a class of antidiabetic drugs initially developed for T2DM management by blocking renal glucose reabsorption (13–15). Intriguingly, SGLT2i have demonstrated significant cardioprotective effects independent of their glucose-lowering properties (16). Clinical studies indicate that these agents improve heart failure symptoms, lowers all-cause and cardiovascular mortality, and reduces the risk of decreased ejection fraction, even in non-diabetic patients (17). Among SGLT2 inhibitors, dapagliflozin has garnered particular attention for its robust cardiovascular benefits (18–20). Mechanistically, dapagliflozin exerts pleiotropic effects by modulating mitochondrial function, reducing oxidative stress, and enhancing ketone body metabolism, which collectively support myocardial energy efficiency and resilience under ischemic conditions (18, 21, 22).

While emerging evidence suggests that dapagliflozin confers significant cardioprotective effects in MI patients, a comprehensive synthesis of clinical outcomes remains lacking, particularly in patients with concurrent T2DM. Here, we systematically evaluate the efficacy of dapagliflozin in MI management, focusing on its impact on cardiac function, heart failure progression, and overall cardiovascular outcomes. Through a meta-analysis of available clinical trials, we establish an evidence-based framework for integrating dapagliflozin into post-MI treatment strategies, with potential implications for both diabetic and non-diabetic patient populations.

## 2 Materials and methods

### 2.1 Search strategy

A comprehensive and systematic literature search was conducted across multiple databases, including PubMed, Embase, Web of Science, Cochrane Library, China National Knowledge Infrastructure (CNKI), and WanFang. The search encompassed all relevant clinical studies, including randomized controlled trials (RCTs) and retrospective cohort studies, published up until May 22, 2024, that investigated the effects of dapagliflozin in myocardial infarction (MI) management. The search terms were as follows: (“dapagliflozin” OR “Farxiga” OR “Forxiga” OR “BMS 512148” OR “BMS512148” OR “BMS-512148”) AND (“Myocardial Infarction” OR “Infarction, Myocardial” OR “Infarctions, Myocardial” OR “Myocardial Infarctions” OR “Cardiovascular Stroke” OR “Cardiovascular Strokes” OR “Stroke, Cardiovascular” OR “Strokes, Cardiovascular” OR “Myocardial Infarct” OR “Infarct, Myocardial” OR “Infarcts, Myocardial” OR “Myocardial Infarcts” OR “Heart Attack” OR “Heart Attacks”). The search strategy was tailored for each database to ensure comprehensive retrieval of relevant studies.

### 2.2 Literature selection and data extraction

Literature selection and data extraction were conducted independently by two researchers based on predefined inclusion and exclusion criteria. Studies were included if (i) they were RCTs and retrospective cohort studies, evaluating the impact of dapagliflozin on MI outcomes investigating the impact of dapagliflozin on MI outcomes; (ii) all patients were diagnosed with a confirmed MI, with or without concurrent T2DM; (iii) studies included a control group receiving standard care for MI, while the intervention group received dapagliflozin as an adjunct to standard care; (iv) studies reported at least one of the following prespecified endpoints, including N-terminal pro-B-type natriuretic peptide (NT-proBNP), left ventricular ejection fraction (LVEF), left ventricular end-diastolic diameter (LVEDD), left ventricular end-systolic diameter (LVESD), high-sensitivity C-reactive protein (hs-CRP), incidence of recurrent myocardial infarction, incidence of heart failure, rate of rehospitalization for heart failure, incidence of stroke, incidence of angina pectoris, and cardiovascular mortality. Studies were excluded if (i) they were cross-sectional studies, case reports, editorials, or reviews; (ii) they involved patient populations without a confirmed MI diagnosis; (iii) they contained insufficient or overlapping data that could not be resolved through correspondence with the authors; (iv) they did not clearly report dapagliflozin administration.

EndNote software was used to manage references and eliminate duplicates. The initial screening was conducted based on titles and abstracts, followed by a full-text review to confirm eligibility. Discrepancies were resolved through consensus or consultation with a third reviewer. Extracted data included information on study author, publication year, patient demographics (age, sex), sample sizes, intervention details, duration of treatment, and outcome indicators, which encompassed NT-proBNP, LVEF, LVEDD, LVESD, hs-CRP, incidence of recurrent MI, heart failure, rehospitalization for heart failure, stroke, angina pectoris, and cardiovascular death.

### 2.3 Statistical analysis

All statistical analyses were performed using Review Manager (RevMan) version 5.3 (The Cochrane Collaboration). Categorical variables were analyzed using relative risk (RR), while continuous outcomes were reported as mean difference (MD) or standardized mean difference (SMD), each with corresponding 95% confidence intervals (CIs). Between-study heterogeneity was evaluated using the I² statistic and associated p-values, with thresholds of I² >50% indicating substantial heterogeneity.

Given the expected methodological and clinical variability across included studies, all meta-analyses were conducted using a random-effects model to ensure robustness, reflect between-study heterogeneity, and maintain conservative estimates of pooled effects. The methodological quality and risk of bias for randomized controlled trials (RCTs) were assessed according to the Cochrane Handbook for Systematic Reviews of Interventions. For retrospective cohort studies, quality was appraised using the Newcastle–Ottawa Scale (NOS).

## 3 Results

### 3.1 Study selection

**Figure 1:**
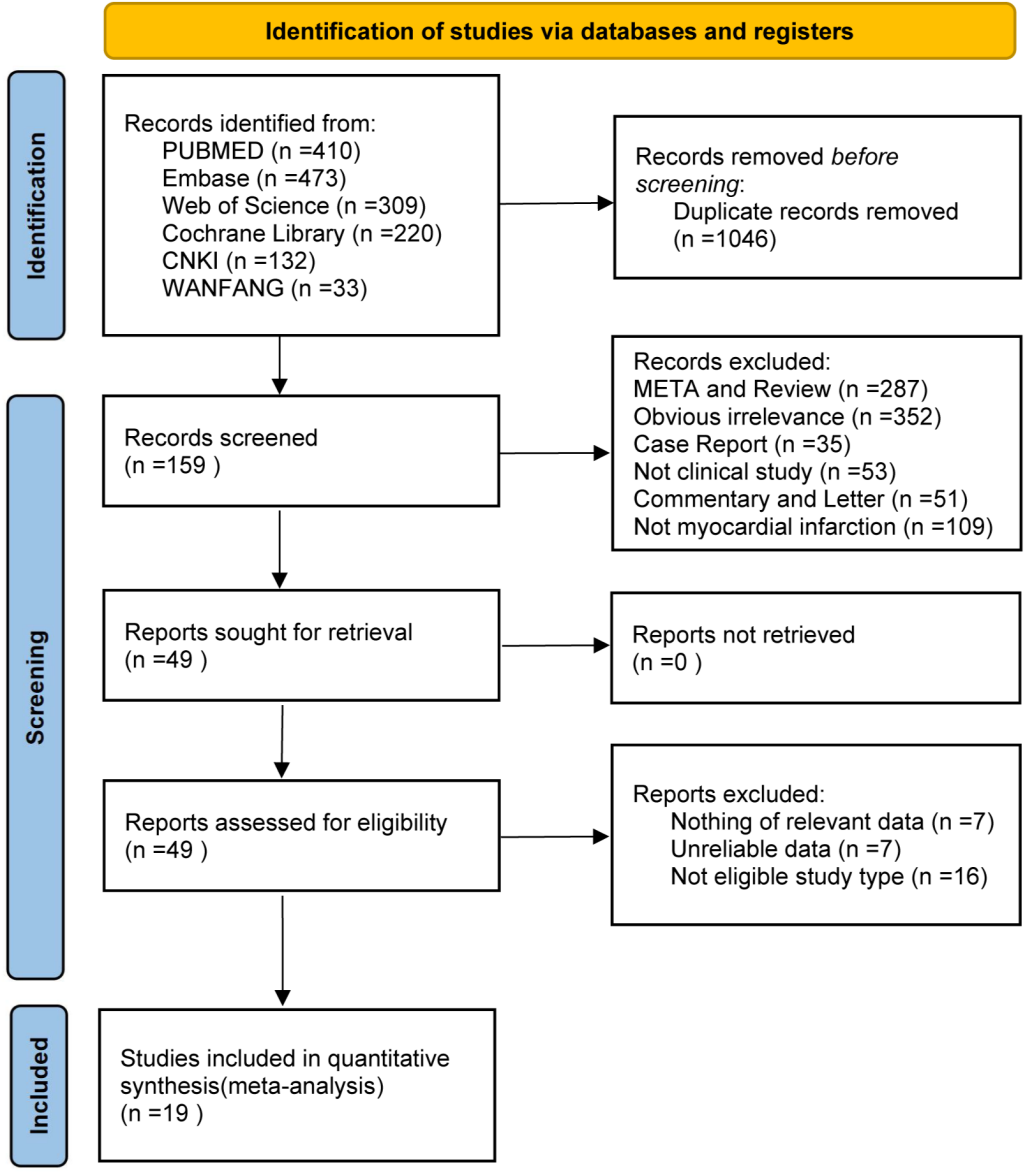
Study selection process for the meta‐analysis of the effect of dapagliflozin on cardiac function and adverse events in myocardial infarction patients.

A comprehensive database (PubMed, Embase, Web of Science, Cochrane Library, CNKI, and WanFang) search identified a total of 1,577 articles. After removing 531 duplicates, 1,046 articles remained for initial screening based on titles and abstracts. Meta-analyses, reviews, case reports, and non-clinical studies were excluded, leaving 159 articles for full-text evaluation. Further exclusions were made due to inconsistent study designs, insufficient data, or unreliable results, culminating in a final selection of 19 studies. The study selection process is illustrated in **Figure 1**.

### 3.2 Overall study characteristics

**Figure 2:**
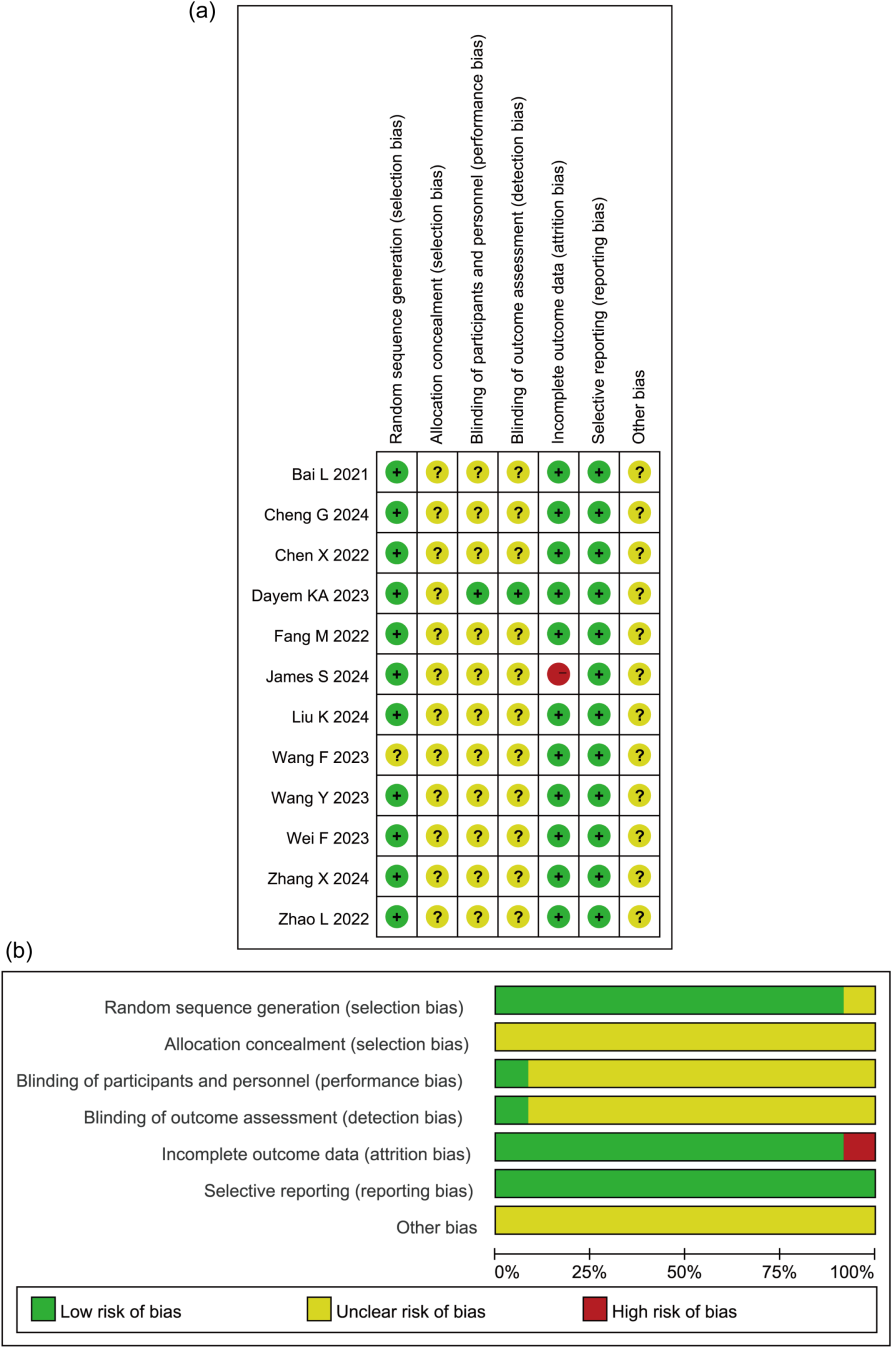
Quality assessment of randomized controlled trials. **(a)** A summary of the basic characteristics of the included studies. **(b)** Proportion of items in methodological quality assessment.

A total of 19 studies, including 7,128 patients with MI, were included in the analysis. Among them, 3,337 patients received dapagliflozin (experimental group), while 3,791 patients received conventional treatment (control group). The characteristics of the included studies are summarized in **Table 1**. Among them, 12 were RCTs, and their methodological quality was assessed using the Cochrane Risk of Bias Tool (Version 5.1.0). As shown in **Figure 2**, all RCTs employed random allocation, with selection bias rated as low risk, although allocation concealment was not explicitly described. One study reported patient dropout due to mortality during the trial, resulting in incomplete outcome data, which was rated as “high risk of bias.” The overall methodological quality of the included RCTs was considered high. For the seven retrospective cohort studies, quality was evaluated using the NOS, with all studies scoring ≥7, indicating high methodological quality (**Table 2**). Given the consistently high quality of both RCTs and retrospective cohort studies, along with the homogeneity in study populations, interventions, control groups, and outcome measures, the results were deemed suitable for pooled analysis.

**Table 1:** The characteristics of the included studies.

ID	Researcher	Year	Observation group	Control group	Included patients	Age, years ± SD		Sex, male/female		BMI(kg/m2)		Hypertension N(%)		Diabetes mellitus N(%)	
						Observation group	Control group	Observation group	Control group	Observation group	Control group	Observation group	Control group	Observation group	Control group
1	Dayem KA (39)	2023	Dapagliflozin 10 mg once daily + GDMT	Placebo: Sugar Tab once daily + GDMT	MI without diabetes	55.24 ± 13.2	56.70 ± 11.5	42/8	41/9	29.96 ± 4.9	30.13 ± 4.6	32 (64.0%)	29 (58.0%)	0 (0%)	0 (0%)
2	James S (40)	2024	10 mg of dapagliflozin daily	placebo	MI without diabetes	63.0±11.06	62.8±10.64	1631/388	1579/419	NA	NA	NA	NA	NA	NA
3	Mao L 41()	2023	oral DAPA 10mg once daily	other kinds of glucose-lowering drugs	MI with diabetes	63.80±12.07	63.80±12.07	181/50	181/50	25.40 (3.33)	24.60 (3.94)	186 (80.5%)	179 (77.5%)	231 (100%)	231 (100%)
4	Zhu Y (22)	2022	DAPA	DAPA-free	MI	60.6±13.6	62.5±13.5	105/36	497/148	26.2±4.1	24.4±3.9	104 (73.8%)	393 (60.9%)	96 (68.1%)	96 (14.9%)
5	Bai L (42)	2021	conventional therapy+oral DAPA 10mg once daily	conventional therapy+other glucose-lowering drugs except SGLT-2i	MI with diabetes	65.60±3.30	65.40±3.20	22/18	23/17	NA	NA	14 (35%)	13 (32.5%)	40 (100%)	40 (100%)
6	Chen X (43)	2022	conventional therapy+DAPA+other glucose-lowering drugs	conventional therapy+other glucose-lowering drugs	MI with diabetes	62.6±12.4	62.9±12.8	NA	NA	NA	NA	74 (59.7%)	73 (59.8%)	124 (100%)	122 (100%)
7	Cheng G (44)	2024	Insulin+DAPA 5-10mg once daily	Insulin	MI with diabetes	55.01±12.94	54.64±12.87	27/13	28/12	NA	NA	NA	NA	40 (100%)	40 (100%)
8	Guo H (45)	2023	dapagliflozin	other glucose-lowering drugs except dapagliflozin	MI with diabetes	67.96±0.78	68.03±0.84	24/16	23/17	NA	NA	NA	NA	40 (100%)	40 (100%)
9	Huo Z (46)	2022	dapagliflozin	other glucose-lowering drugs except SGLT-2i	MI with diabetes	61.3±9.7	63.4±9.8	112/51	90/48	26. 33±3. 46	26. 34±3. 47	115( 70. 6)	102( 73. 9)	163 (100%)	138 (100%)
10	Jiang X (47)	2022	dapagliflozin	other glucose-lowering drugs except SGLT-2i	MI with diabetes	68.3±8.5	65.9±5.6	15/6	14/7	NA	NA	NA	NA	21 (100%)	21 (100%)
11	Liu K (48)	2024	conventional therapy+DAPA 5-10mg once daily	conventional therapy	MI with diabetes	62.72±7.61	64.40±6.94	44/11	37/16	24. 17±2. 51	24. 75±2. 49	NA	NA	55 (100%)	53 (100%)
12	Wei F (49)	2023	oral DAPA 10mg once daily	Insulin	MI with diabetes	61.48±11.65	64.12±10.89	32/10	30/12	NA	NA	NA	NA	42 (100%)	42 (100%)
13	Yang C (50)	2023	10mg dapagliflozin daily	other glucose-lowering drugs except SGLT-2i	MI with diabetes	60.89±10.65	59.27 ±11.01	68/42	72/38	24.73±3.64	25.14± 3.39	81(73.64)	78(70.90)	110 (100%)	110 (100%)
14	Yin Y (51)	2021	Metformin+dapagliflozin	Metformin	MI with diabetes	47.51±12.57	48.03±11.69	16/14	15/15	27.05±1.43	26.69±1.67	NA	NA	30 (100%)	30 (100%)
15	Zhao L (52)	2022	Insulin+DAPA 5-10mg once daily	Insulin	MI with diabetes	59.67±8.64	59.38±8.05	33/16	31/18	27.55±2.24	27.49±2.42	NA	NA	49 (100%)	49 (100%)
16	Wang Y (53)	2023	conventional therapy+dapagliflozin 10mg qd	conventional therapy	MI without diabetes	69.31±3.46	68.58±3.94	24/21	23/22	NA	NA	43(95.56)	40(88.89)	0 (0%)	0 (0%)
17	Zhang X (54)	2024	metoprool +conventional treatment +dapagliflozin	metoprool+ conventional treatment	MI	56.91±6.58	57.62±6.82	26/17	23/20	22.79±2.83	22.41±2.78	18 (41.9)	21 (48.8)	10 (23.3%)	8 (18.6%)
18	Wang F (55)	2023	conventional treatment +dapagliflozin	conventional treatment	MI	59.3 ± 8.37	56.8 ± 9.14	29/5	30/4	24.3 ± 6.78	24.7 ± 5.83	16(47)	15(44)	6(18)	9(26)
19	Fang M (56)	2022	conventional treatment +dapagliflozin 10mg daily	conventional treatment	MI	60.59 ±5.37	60.60 ± 5.31	35/25	32/28	NA	NA	42(70.00)	45(75.00)	NA	NA

NA, not available.

**Table 2:** Quality assessment of the included studies by the Newcastle–Ottawa scale.

Study	Year	Design	Selection	Comparability	Outcome	Score
Mao L (41)	2023	Retrospective cohort	★★★	★★	★★★	8
Zhu Y (22)	2022	Retrospective cohort	★★★★	★★	★★★	9
Guo H (45)	2023	Retrospective cohort	★★★	★	★★★	7
Huo Z (46)	2022	Retrospective cohort	★★★	★★	★★★	8
Jiang X (47)	2022	Retrospective cohort	★★★	★★	★★★	8
Yang C (50)	2023	Retrospective cohort	★★★	★★	★★★	8
Yin Y (51)	2021	Retrospective cohort	★★★	★	★★★	7

★equals one point, with a total of 10 points.

### 3.3 Dapagliflozin in improving cardiac function in MI patients

#### 3.3.1 NT-proBNP

**Figure 3:**
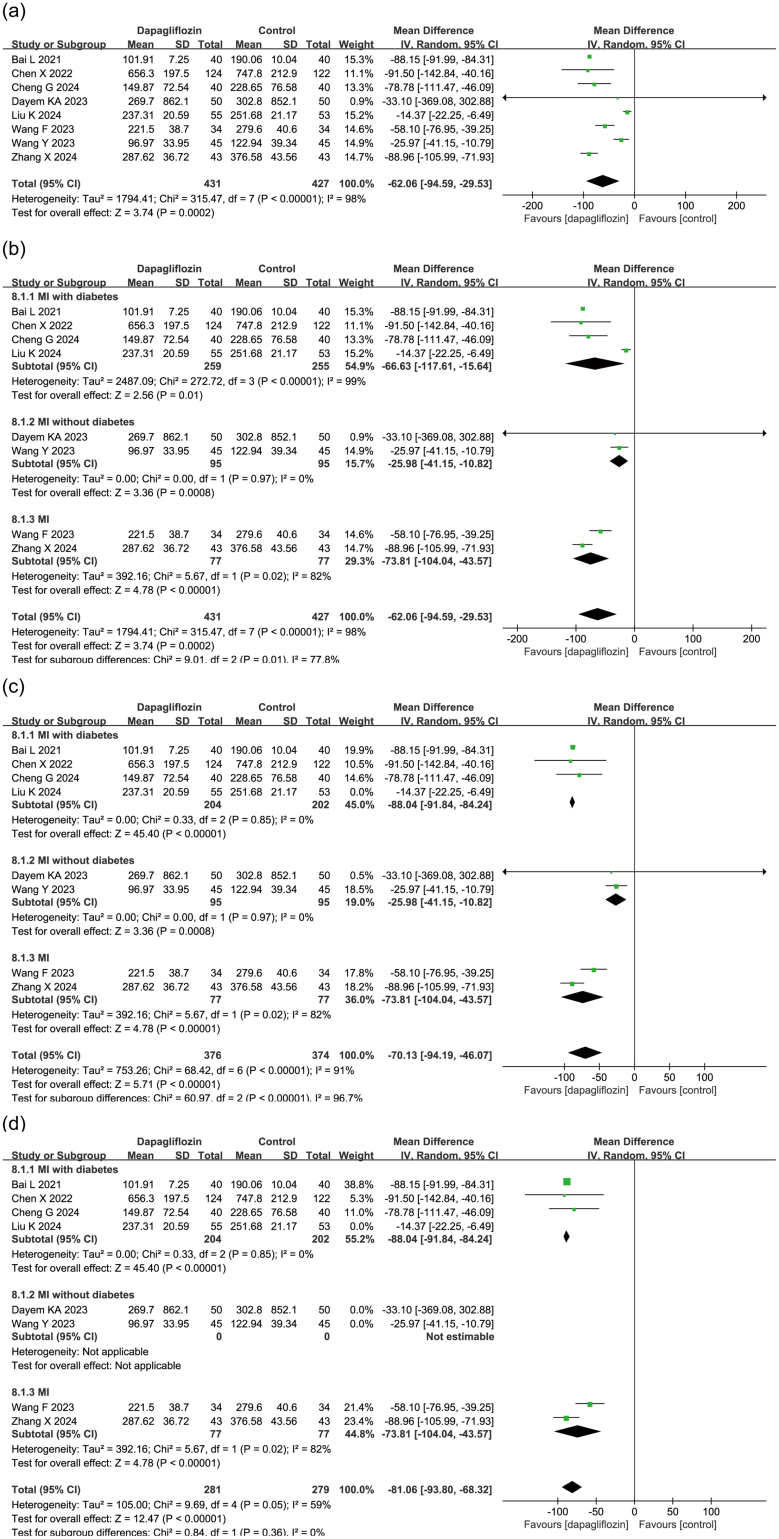
Forest plot of dapagliflozin treatment on NT-proBNP in myocardial infarction patients. **(a)** Forest plot summarizing the impact of dapagliflozin treatment on NT-proBNP levels in patients with myocardial infarction. **(b)** Subgroup analysis based on diabetes status, showing NT-proBNP outcomes in patients with and without diabetes. **(c)** Sensitivity analysis conducted by sequentially excluding individual studies; the study by Liu et al. was identified as a major source of heterogeneity. Forest plot after removing Liu et al.'s study is presented. **(d)** Sensitivity analysis excluding the entire non-diabetic subgroup, which markedly reduced heterogeneity, indicating that diabetes status is a key contributor to between-study variability.

8 studies (n = 858) evaluated the effect of dapagliflozin on NT-proBNP levels, a biomarker of myocardial stress. Due to high heterogeneity (I² = 98%), a random-effects model was applied, revealing a significant reduction in NT-proBNP levels in the dapagliflozin group compared to controls (MD = -62.06, 95% CI [-94.59, -29.53], P = 0.0002) (**Figure 3a**).

To explore sources of heterogeneity, a subgroup analysis was performed based on diabetes status. Among the included studies, 4 studies focused on patients with diabetes (MI with diabetes), 2 studies on non-diabetic patients (MI without diabetes), and 2 did not distinguish diabetes status. **Figure 3b** shows that heterogeneity remained high within the MI with diabetes and MI subgroups, but all subgroups consistently demonstrated a significant NT-proBNP-lowering effect in the dapagliflozin group.

A sensitivity analysis was conducted by sequentially excluding each study. The study by Liu et al. in the MI with diabetes subgroup was identified as a major source of heterogeneity (**Figure 3c**). Additionally, removing the entire MI without diabetes subgroup significantly reduced heterogeneity, indicating that diabetes status was a key contributor to heterogeneity (**Figure 3d**).

#### 3.3.2 Left ventricular ejection fraction

**Figure 4:**
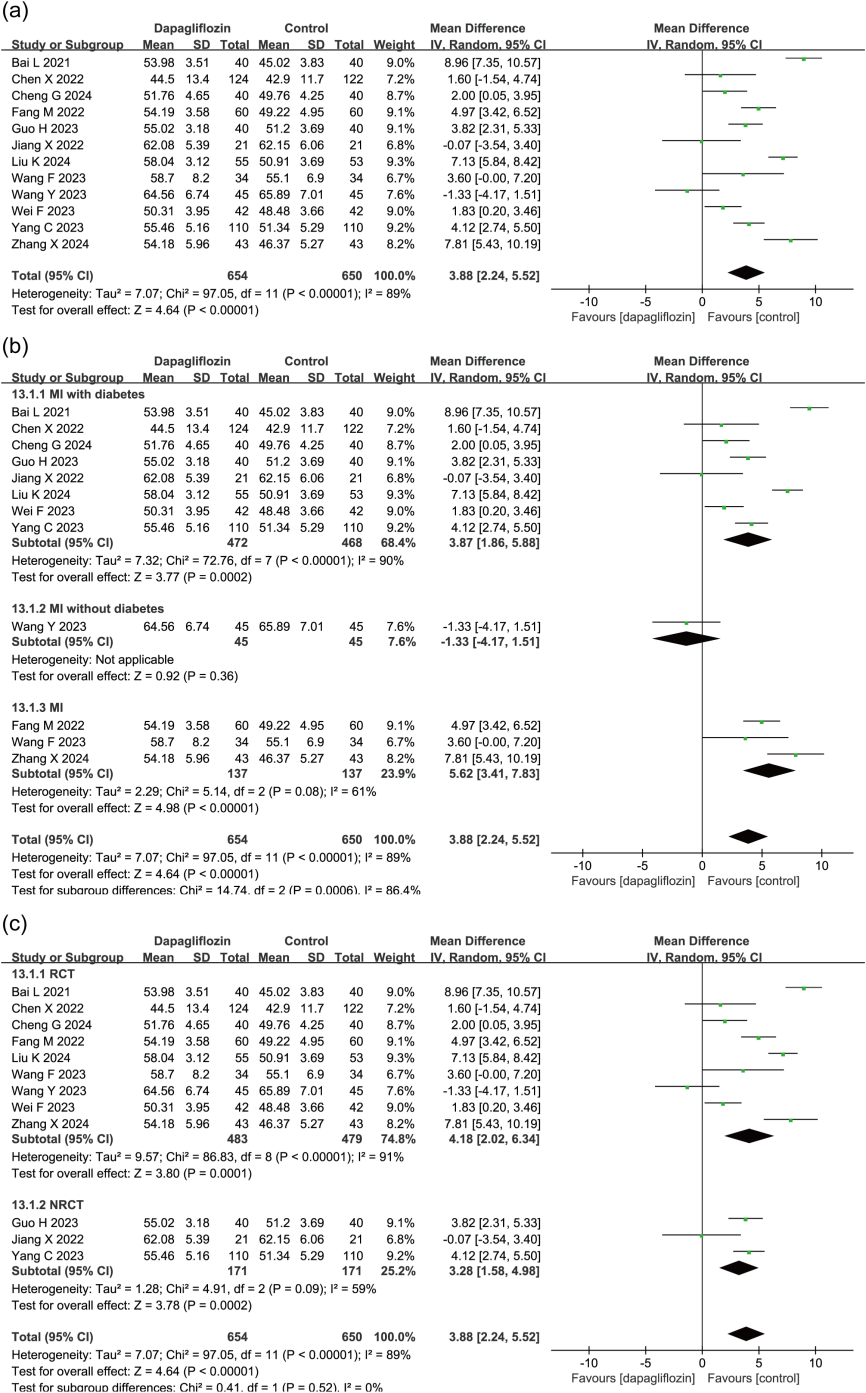
Forest plot of dapagliflozin treatment on LVEF in myocardial infarction patients. **(a)** Overall effect on LVEF. **(b)** Subgroup analysis stratified by diabetes status. **(c)** Subgroup analysis comparing randomized controlled trials (RCTs) and non-randomized controlled trials (NRCTs).

12 studies (N = 1304) evaluated the impact of dapagliflozin on LVEF in patients with myocardial infarction, including 654 patients in the dapagliflozin group and 650 in the control group. Meta-analysis using a random-effects model demonstrated a significant improvement in LVEF in the dapagliflozin group compared to controls (MD = 3.88, 95% CI: 2.24–5.52, P < 0.00001) (**Figure 4a**).

Given the observed heterogeneity, subgroup analysis was conducted based on diabetes status (**Figure 4b**). Of the 12 studies, 8 enrolled patients with T2DM, 1 enrolled non-diabetic patients, and 3 did not specify diabetes status. In the T2DM and unspecified cohorts, dapagliflozin treatment was associated with significantly greater improvements in LVEF compared to control. The single non-diabetic study showed no significant difference.

To further assess potential variability between study designs, subgroup analysis was performed based on study type. Among the 12 included studies, 9 were RCTs and 3 were NRCTs. Subgroup analysis revealed consistent results across both RCT and NRCT subgroups, each demonstrating statistically significant improvements in LVEF with dapagliflozin, supporting the robustness of the pooled effect (**Figure 4c**).

#### 3.3.3 Left ventricular end-diastolic diameter

**Figure 5:**
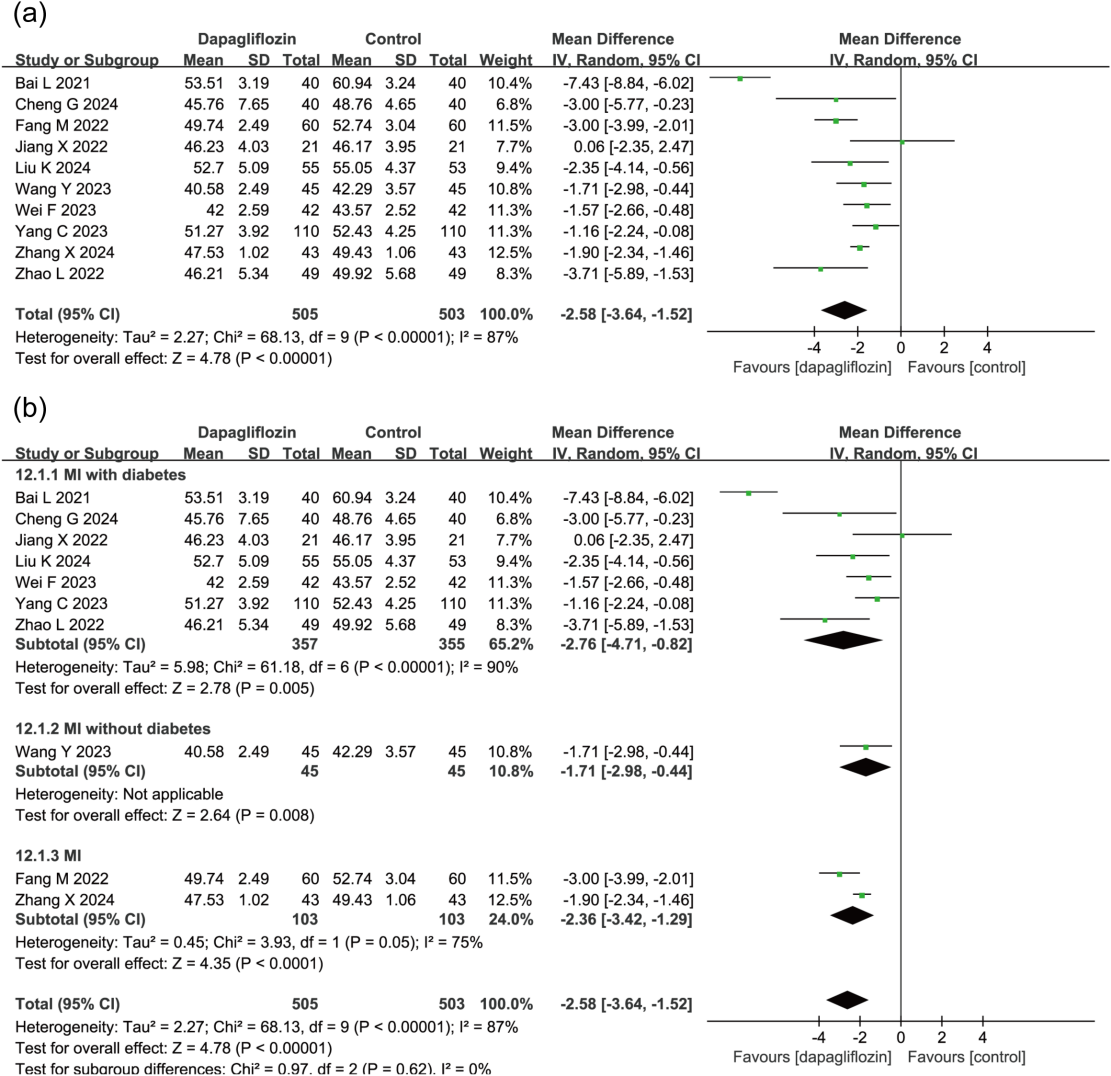
Forest plot of dapagliflozin treatment on LVEDD in myocardial infarction patients. **(a)** Forest plot illustrating the effect of dapagliflozin treatment on LVEDD in patients with myocardial infarction. **(b)** Subgroup analysis forest plot of LVEDD stratified by diabetes status, comparing outcomes in diabetic and non-diabetic patients.

10 studies (n = 1,008) assessed LVEDD, revealing a significant reduction in the dapagliflozin group (MD = -2.58, 95% CI [-3.64, -1.52], P < 0.00001) using a random-effects model due to high heterogeneity (I² = 87%) (**Figure 5a**). Subgroup analysis based on diabetes status indicated a consistent and statistically significant reduction in LVEDD across all subgroups (**Figure 5b**).

#### 3.3.4 Left ventricular end-systolic diameter

**Figure 6:**
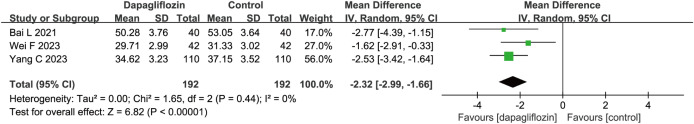
Forest plot of dapagliflozin treatment on LVESD in myocardial infarction patients. LVESD, left ventricular end- systolic diameter.

3 studies (n = 384) demonstrated a significant reduction in LVESD in the dapagliflozin group (MD = -2.32, 95% CI [-2.99, -1.66], P < 0.00001). Notably, no heterogeneity was detected (P = 0.44, I² = 0%) (**Figure 6**), supporting a robust and consistent benefit of dapagliflozin in reducing LVESD.

### 3.4 Dapagliflozin in reducing adverse cardiovascular events in patients with MI

#### 3.4.1 Cardiovascular death

**Figure 7:**
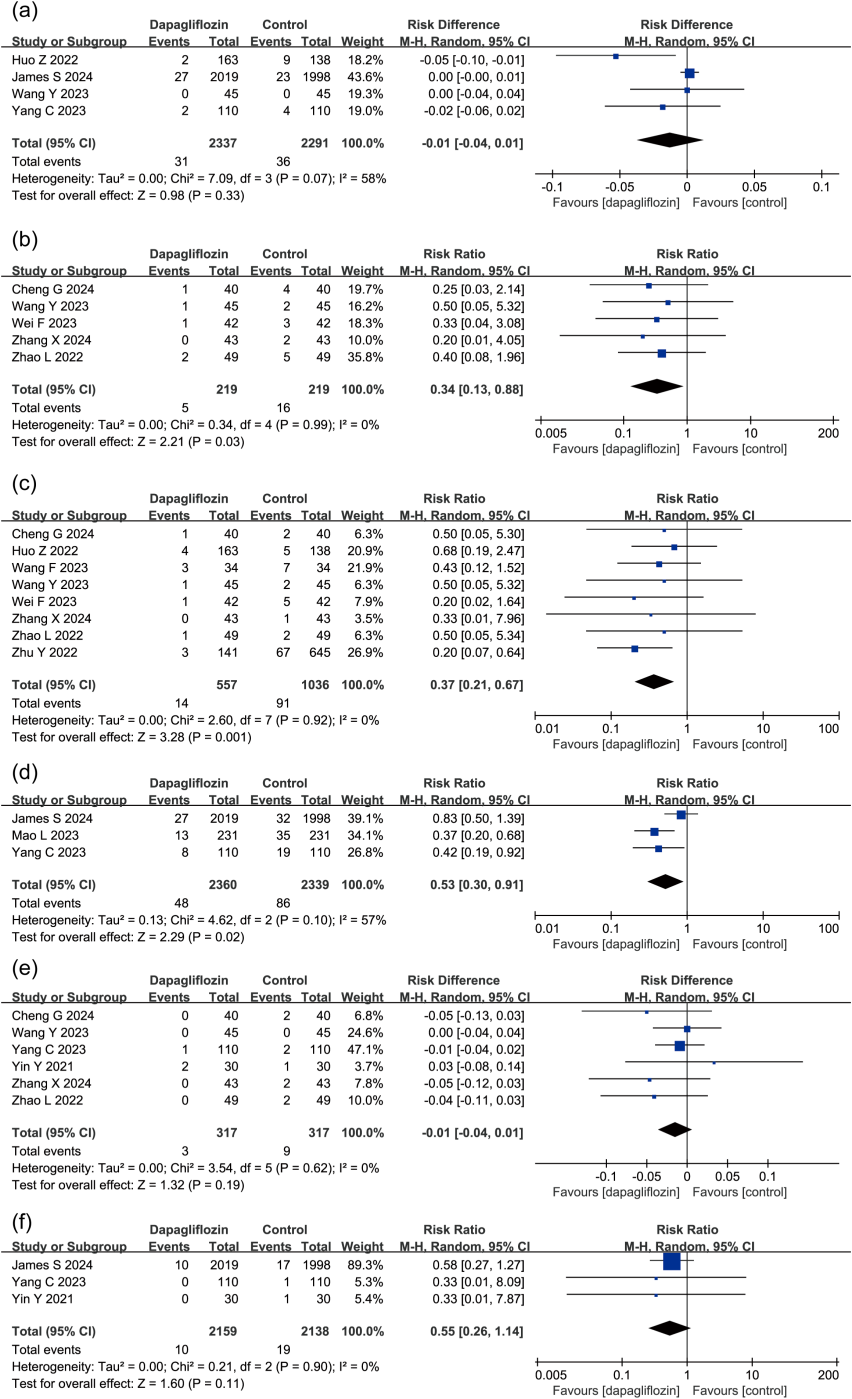
Forest plot of dapagliflozin treatment on **(a)** CV death, **(b)** Angina Pectoris, **(c)** incidence of heart failure, **(d)** heart failure rehospitalization rate, **(e)** recurrent myocardial infarction, **(f)** stroke in myocardial infarction patients. CV death, cardiovascular death.

4 studies (n = 4,628) assessed CV death (2,337 dapagliflozin; 2,291 control). In one study (Wang et al.), both experimental and control groups reported zero CV death events, preventing relative risk (RR) calculation. Therefore, risk difference (RD) was used as the effect size. Due to moderate heterogeneity (P = 0.07, I² = 58%), a random-effects model was applied, showing no statistically significant reduction in CV death (P = 0.33) (**Figure 7a**). These findings suggest that current evidence is insufficient to confirm dapagliflozin’s effect on post-MI CV death incidence.

#### 3.4.2 Angina pectoris

5 studies (n = 438) evaluated angina pectoris risk, with 219 patients per group. Dapagliflozin significantly reduced angina pectoris incidence compared to controls (RR = 0.34, 95% CI [0.13, 0.88], P < 0.05) (**Figure 7b**).

#### 3.4.3 Heart failure

8 studies (n = 1,593) analyzed heart failure incidence (557 dapagliflozin; 1,036 control), a random-effects model revealing a significant reduction in heart failure risk in the dapagliflozin group (RR = 0.37, 95% CI [0.21, 0.67], P < 0.05) (**Figure 7c**).

#### 3.4.4 Rehospitalization for heart failure

3 studies (n = 4,699) evaluated rehospitalization due to heart failure (2,360 dapagliflozin; 2,339 control). Moderate heterogeneity was observed (P = 0.1, I² = 57%), necessitating a random-effects model. The analysis showed a significant reduction in rehospitalization rates in the dapagliflozin group (RR = 0.53, 95% CI [0.30, 0.91], P < 0.05) (**Figure 7d**).

#### 3.4.5 Recurrent MI

6 studies assessed MI recurrence in 634 patients. In Wang et al., both groups had zero MI events, making RR calculation impossible. Thus, RD was used as the effect size, a random-effects model showing no significant effect (P = 0.19) (**Figure 7e**), suggesting dapagliflozin did not significantly reduce recurrent MI risk.

#### 3.4.6 Stroke

3 studies (n = 4,297) examined stroke incidence, with no statistically significant effect observed (P > 0.05), suggesting dapagliflozin may not reduce stroke risk in MI patients (**Figure 7f**).

**Figure 8:**
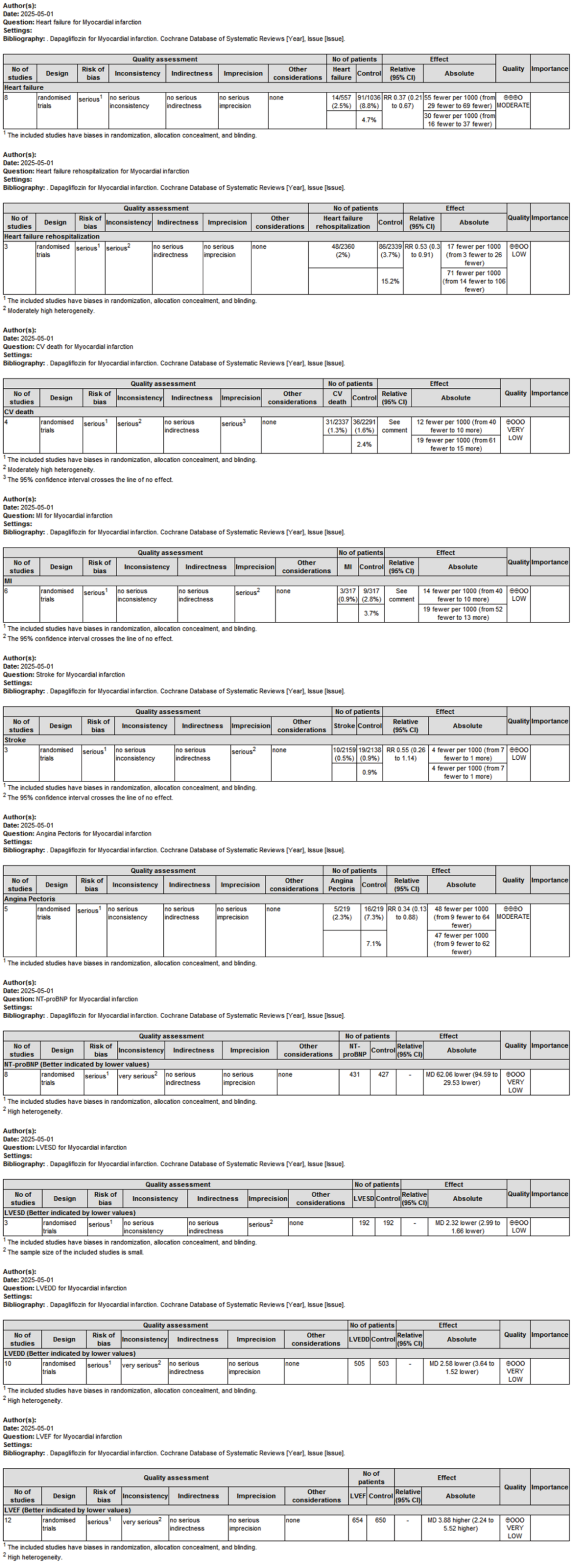
GRADE evidence profile evaluating the overall certainty of evidence across outcomes related to dapagliflozin treatment in MI patients.

To systematically evaluate the quality of evidence across included studies and provide reliable recommendations for clinical practice, the Grading of Recommendations Assessment, Development and Evaluation (GRADE) framework was applied (**Figure 8**). The overall certainty of evidence ranged from moderate to low, primarily due to heterogeneity among studies and limitations in study design.

## 4 Discussion

Despite dapagliflozin’s established benefits in reducing cardiovascular risk among patients with heart failure, its specific impact on outcomes in MI patients has not been comprehensively analyzed (23, 24). This gap is clinically significant, as MI presents unique pathophysiological challenges distinct from other cardiovascular conditions (25). Here, we conduct a systematic evaluation of dapagliflozin’s effects on cardiac function indicators, including NT-proBNP, LVEF, LVEDD, and LVESD (26), and the incidence of adverse cardiovascular outcomes such as angina pectoris, heart failure, and rehospitalization due to heart failure. Our findings provide critical insights into the potential therapeutic benefits of dapagliflozin for improving prognosis in this high-risk group, offering a foundation for future targeted clinical strategies.

Our data indicate that dapagliflozin effectively reduces NT-proBNP and hs-CRP levels, while increasing LVEF and decreasing LVEDD and LVESD. NT-proBNP, a natriuretic peptide, is a highly regarded biomarker for heart failure and a prognostic indicator in cardiovascular diseases (27, 28). Elevated NT-proBNP levels are associated with increased heart stress and adverse outcomes (29); thus, the observed reduction suggests dapagliflozin may alleviate myocardial stress post-MI. Elevated LVEF indicates improved left ventricular function (30), suggesting that dapagliflozin could enhance the heart’s capacity to pump blood. Since the left ventricle is crucial for systemic oxygen distribution, improvements in these indicators underscore the drug’s potential to support myocardial recovery and mitigate heart failure risk.

Heart failure is a common and severe complication following MI, with incidence rates between 14% and 36% among acute MI patients (31, 32). The results of this meta-analysis show that dapagliflozin significantly reduces the occurrence of heart failure events and decreases rehospitalization rates for heart failure. The reduction in heart failure events, along with improvements in NT-proBNP and LV function markers, suggests dapagliflozin may play a protective role against the progression to heart failure. Additionally, dapagliflozin was associated with a lower incidence of angina pectoris, underscoring its potential to reduce myocardial ischemic episodes. However, the effects on recurrent MI, stroke, and cardiovascular (CV) death were not statistically significant, indicating that while dapagliflozin may be beneficial in some areas, further evidence is needed to clarify its impact on these endpoints, in agreement with published results (33, 34).

The beneficial effects of dapagliflozin on cardiac function and heart failure prevention may be due to several mechanisms beyond glycemic control. Dapagliflozin, an SGLT2 inhibitor, promotes diuresis and natriuresis, which reduces preload and afterload, thereby lowering myocardial workload (35, 36). Additionally, SGLT2 inhibitors have been shown to improve myocardial metabolism by enhancing ketone body utilization, which may be more energy-efficient for the heart under stress (37). The observed reduction in hs-CRP, a marker of systemic inflammation (38), suggests dapagliflozin may also reduce inflammation—a key factor in post-MI remodeling and heart failure progression. These multifaceted effects could collectively contribute to the observed improvements in cardiac function and reduction in certain cardiovascular events.

While this meta-analysis provides valuable insights, certain limitations must be acknowledged. First, heterogeneity in the concomitant medications used in the various studies may have influenced outcomes. Variations in conventional MI treatments, such as differences in beta-blocker or ACE inhibitor use, could introduce confounding effects that impact the analysis. Second, the inclusion of only four English-language studies, with the majority of data derived from Chinese literature, limits the generalizability of the findings. Some of the included studies had small sample sizes, which may affect the robustness of the pooled results. Lastly, the overall methodological quality of the included studies was moderate. Many studies lacked detailed descriptions of blinding and allocation concealment, potentially increasing the risk of bias and impacting the strength of the evidence.

Our results suggest that dapagliflozin, in conjunction with conventional treatment, offers promising benefits in improving cardiac function and reducing specific adverse cardiovascular events in MI patients. Given the observed reductions in NT-proBNP and improvements in LVEF, dapagliflozin could be considered as an adjunct therapy for MI patients, especially those at high risk of heart failure. However, the non-significant effects on recurrent MI, CV death, and stroke highlight the need for further research to define dapagliflozin’s full therapeutic potential.

Future studies should focus on larger, multicenter RCTs with rigorous methodological designs, including standardized use of conventional therapies and clear descriptions of blinding and allocation procedures. Additionally, longer follow-up periods would be valuable for assessing the long-term impact of dapagliflozin on recurrent MI and mortality. By addressing these gaps, future research can provide stronger evidence on dapagliflozin’s role in comprehensive post-MI management.

## 5 Conclusion

Dapagliflozin appears to improve key cardiac function parameters and reduce heart failure-related outcomes in MI patients, supporting its potential role in post-MI therapy. Further high-quality studies are essential to confirm these findings and guide clinical application.

## Data Availability

The original contributions presented in the study are included in the article/supplementary material. Further inquiries can be directed to the corresponding author.
